# A Paraneoplastic Syndrome Characterized by Extremity Swelling with Associated Inflammatory Infiltrate Heralds Aggressive Transformation of Myelodysplastic Syndromes/Myeloproliferative Neoplasms to Acute Myeloid Leukemia: A Case Series

**DOI:** 10.1155/2012/582950

**Published:** 2012-06-25

**Authors:** James K. Mangan, Selina M. Luger

**Affiliations:** Division of Hematology-Oncology, Department of Medicine, Abramson Cancer Center, University of Pennsylvania, 34th Street and Civic Center Blvd, Philadelphia, PA 19104, USA

## Abstract

There has been a long history of reports describing a variety of paraneoplastic phenomena associated with myelodysplastic syndromes, particularly those with autoimmune manifestations. We report here a series of patients with an antecedent myelodysplastic syndrome (MDS) or myeloproliferative neoplasm (MPN) that underwent aggressive transformation to acute myeloid leukemia (AML). In each case, the transformation to AML was preceded by an inflammatory syndrome characterized by unilateral extremity swelling and an associated inflammatory skin infiltrate, as well as other signs of inflammation, including profound hyperferritinemia without evidence of a hemophagocytic syndrome. We suggest that such an inflammatory syndrome may herald aggressive transformation of MDS/MPN to AML. Patients with known MDS/MPN who present with these features may benefit from early bone marrow examination to assess disease status. Early intervention with corticosteroids in select patients may result in improvement or resolution of the symptoms and permit intensive therapy for AML to be delivered.

## 1. Introduction

Myelodysplastic syndromes and myeloproliferative neoplasms have a variable course. Some exhibit indolent behavior over several years or the course of a lifetime, while others transform to acute myeloid leukemia. For MDS, the International Prognostic Scoring System (IPSS) is a well-validated model used to predict overall survival and risk of transformation to AML [1]. Likewise, the lifetime risk of transformation to acute leukemia for MPNs such as polycythemia vera (PV) and essential thrombocytosis (ET) is well established and an IPSS and cytogenetics have been used to assess survival and risk of transformation to acute leukemia in primary myelofibrosis [2]. Although the likelihood of transformation to acute myeloid leukemia is predictable, the precise timing of disease progression can vary. Additionally, many patients with MDS or MPN do not come to clinical attention until they have transformed to AML.

We describe here a series of three patients, each of whom had an antecedent bone marrow disorder before transforming to an acute myeloid leukemia with an aggressive clinical course. Prior to transformation to AML, the patients became ill with a syndrome characterized by unilateral extremity swelling with an associated inflammatory infiltrate as well as hyperferritinemia without evidence of a hemophagocytic syndrome. It appears that the inflammatory syndrome heralded transformation to a clinically aggressive acute myeloid leukemia in each case.

## 2. Case Presentations

**Patient 1**

Patient 1 is a 65-year-old man with a history of antecedent low-risk MDS who underwent a bone marrow biopsy one year later that was concerning for chronic myelomonocytic leukemia. The marrow was hypercellular (80–90%) with 7–10% blasts, and cytogenetics demonstrated del 5q and trisomy 8. CBC at the time showed a WBC count of 25,500/*μ*L (16% neutrophils, 49% lymphocytes, and 35% “mid” forms), hemoglobin 7.7 g/dL, and platelets 47,000/*μ*L. The patient was started on 5-azacitadine shortly after this bone marrow biopsy but only received one cycle before he was admitted with neutropenic fever. While he was being treated for an indwelling venous catheter infection, he developed marked left lower extremity swelling with 3+ edema. He also had massive testicular edema. The patient underwent a Doppler ultrasound of his left lower extremity that revealed no clot. An MRI of the patient's left lower extremity revealed a large mass involving the medial upper thigh, 16 × 9 × 11 centimeters, extending into the inferior aspect of the left hip. It was felt to be compatible with large abscess formation or chronic hematoma. The patient was taken to surgery for exploration and drainage of the thigh wound but no infection was found. A drain was placed but was later removed because there was no significant drainage. Biopsy of the left thigh showed congested and edematous soft tissue with scattered atypical mononuclear cells, which were not felt to adequately explain the mass seen clinically. It was felt that the presence of atypical mononuclear cells was more likely to represent peripheral blood trafficking than a granulocytic sarcoma. The patient was discharged after a month-long hospitalization and presented to our institution as an outpatient.

Upon presentation to our institution, the patient was noted to now have swelling of the contralateral (right) lower extremity and 3+ edema from his right foot to right thigh. There was induration on the medial aspect of his right groin and he could not stand up on his own because of leg pain. Complete blood count with differential was as follows: WBC 5,900/*μ*L (20% neutrophils, 17% bands, 32% monocytes, 6% myelocytes, 2% promyelocytes, 3% blasts) hemoglobin 9.6 g/dL and platelets 52,000/*μ*L. He was admitted to the hospital with extremity swelling concerning for cellulitis versus deep venous thrombosis (DVT).

Doppler ultrasound of his right lower extremity showed no evidence of DVT. He was started empirically on antibiotics for cellulitis, but further investigation of the area of induration on his right thigh by punch biopsy demonstrated an inflammatory infiltrate more consistent with urticaria (focal spongiosis, superficial dermal edema and sparse perivascular lymphocytic infiltrate with mast cells and rare eosinophils). Purpuric lesions on the left thigh were shown to be consistent with leukocytoclastic vasculitis. Magnetic resonance imaging (MRI) of the right lower extremity and pelvis to further evaluate for abscess or fluid collection as a cause of the patient's right lower extremity swelling revealed only diffuse soft tissue edema consistent with anasarca.

Laboratory studies on admission were notable for a ferritin of 13,520 ng/mL and an albumin of 1.4 g/dL. The ferritin was too high to be explained solely by the estimated >40 units of blood the patient had been transfused in the past year. Careful examination of a repeat bone marrow biopsy showed no evidence of hemophagocytic syndrome, so the high ferritin was felt to be reactive.

Hypoalbuminemia was sufficient to explain his impressive scrotal edema and overall anasarca. 24-hour urine protein was 2.0 grams per total volume. His overall medical condition was felt to be too tenuous for renal biopsy to further characterize the nature of his protein losing nephropathy. He demonstrated mild renal insufficiency but did not develop acute kidney injury.

A bone marrow biopsy was performed to assess for disease transformation. This showed an expansion of blasts (37%) with monocytic features consistent with AML M5 by French-American-British (FAB) classification or AML with myelodysplasia related changes by World Health Organization (WHO) classification. FLT3 mutation analysis was negative but the patient had a complex karyotype with more than ten cytogenetic abnormalities.

The patient developed left elbow swelling with limited range of motion and a series of tests to look for rheumatologic or infectious causes of inflammation were run, but all were negative (ANA, antimitochondrial antibodies, ANCA, cryoglobulins, and HIV). A brief course of prednisone did not lead to improvement in his extremity swelling, anasarca, or nephropathy. His poor performance status precluded standard induction chemotherapy for AML or enrollment on a clinical trial, and the patient ultimately developed pneumonia with increasing oxygen requirement and associated sepsis. A decision was made to provide comfort measures rather than pursuing aggressive supportive care in the intensive care unit and the patient passed away 30 days into his hospitalization.

**Patient 2**

The second patient is a 54-year-old woman who initially presented with fatigue. She also noticed an unusual swelling of her right calf. She visited her primary care physician and underwent a workup to look for causes of her pronounced malaise. A mild anemia (Hbg 10.5 g/dL) was noted but the patient's other blood counts were normal. Fatigue workup also included negative tests for Lyme disease and influenza. Later that month, she was admitted to her local hospital with abdominal pain and diarrhea. She was diagnosed with an ileitis and tests for *C. difficile* were negative. She was noted to have a significant leukocytosis (WBC 18,000/*μ*L) with left shift (72% neutrophils, 13% bands), continued anemia (Hbg 9.1 g/dL), and Plts 325,000/*μ*L. After 4-5 days of empiric metronidazole therapy, the patient was discharged with improved abdominal pain.

The patient was readmitted shortly thereafter, however, with a swollen and erythematous left elbow. An incision and drainage at the site of the elbow erythema was performed, but all cultures were negative and the joint itself was not infected. The patient was treated with IV antibiotics for cellulitis and discharged on oral antibiotics. Her primary care physician examined her twice during the following week, both times with a swollen, painful right thigh. Doppler ultrasound showed no evidence of DVT. Several days later, her right thigh became warm and erythematous. CBC demonstrated a WBC count of 46,000/*μ*L with increased bands but no blasts, Hgb 9.7 g/dL, Plts 190,000/*μ*L. She was readmitted to the hospital with a diagnosis of cellulitis. In addition, she was experiencing significant diarrhea and had developed frank anasarca. She was transferred to the medicine service at our institution two months after her initial presentation, with ongoing anasarca, right leg pain and swelling, intermittent fevers, and failure to thrive.

MRI of her right leg showed changes consistent with pyomyositis. The patient's leg was therefore drained but cultures were negative and she was ultimately converted from intravenous to oral antibiotics. Laboratory studies were notable for a ferritin that was markedly elevated (6,165 ng/mL), consistent with an inflammatory syndrome.

The patient's anasarca initially progressed, with albumin below the detectable limits of the assay. Nephrotic syndrome was ruled out and the diagnosis of protein losing enteropathy was entertained, but colonoscopy and esophagogastroduodenoscopy (EGD) did not support this.

Left-shifted leukocytosis, anemia, and thrombocytopenia persisted during her hospitalization. A bone marrow biopsy was performed which showed a markedly hypercellular marrow (>90%) with significant myeloid hyperplasia, hypersegmented neutrophils, erythroid hypoplasia, and megakaryocytic dysplasia. The differential diagnosis was felt to include a reactive process in the setting of the patient's worsening overall condition versus a myeloproliferative neoplasm. The significant megakaryocytic atypia was felt to favor a hematopoetic neoplasm, and the possibility of primary myelofibrosis was raised because of significant reticulin fibrosis that was seen. Cytogenetics were normal and RT-PCR for BCR-ABL and Jak2 V617F from the peripheral blood was negative.

In the setting of ongoing anasarca and hypoalbuminemia with no clear underlying cause, an empiric course of high-dose steroids was started following the completion of the bone marrow biopsy. With steroids, the patient's anasarca and hypoalbuminemia gradually improved, and she was discharged to a rehabilitation facility. Steroids were gradually tapered to 15 mg daily over the course of the next three months.

Routine CBC three months after discharge then surprisingly demonstrated a WBC count of >40,000/*μ*L with 50% myeloblasts detected by flow cytometry. Subsequent bone marrow biopsy confirmed the diagnosis of AML, WHO category acute panmyelosis with myelofibrosis. Cytogenetics were normal but the patient expressed an FLT3 internal tandem duplication mutation. She was treated with mitoxantrone, etoposide, and cytarabine chemotherapy and achieved complete remission. Steroids were tapered to off.

With successful treatment of her leukemia, the patient returned to excellent functional status. Unfortunately, her leukemia relapsed shortly prior to planned admission for allogeneic stem cell transplant. She was refractory to salvage chemotherapy but did achieve a complete remission without platelet recovery after 40 days of therapy with a novel FLT3 inhibitor and was able to undergo fully myeloablative allogeneic stem cell transplant from her HLA identical brother. However, the patient relapsed with high WBC count on day +80 after transplant and expired 30 days later with complications from progressive AML.

**Patient 3**

The third patient is a 75-year-old man who was initially diagnosed with a low-risk myelodysplastic syndrome three years prior to transformation to acute myeloid leukemia. Upon initial presentation, he was found to have a hypercellular bone marrow (65%) with left-shifted hematopoiesis and subtle dysplasia, with a normal karyotype and no excess blasts. He underwent treatment with pegfilgrastim and darbopoietin then underwent therapy with ten cycles of hypomethylating agents before losing his response and beginning therapy with lenalidomide. During lenalidomide therapy, the patient required two units of packed red blood cells every 7–10 days. After being on lenalidomide for several months, the patient developed an area of erythema on his left knee, which became progressively more swollen. He was admitted to the hospital for surgery and the mass was removed. Pathology demonstrated chronic fibrosing panniculitis with central acutely inflamed abscess, fat necrosis, and acute and chronic hemorrhage as well as recanalized chronic venous thrombosis. No leukemic infiltrates were seen, and there was no evidence of malignancy. The patient was discharged home following his surgery. Blood counts at this time showed stable pancytopenia. After several weeks at home, the patient was hospitalized for complications from his nonhealing left knee wound and required a wound vacuum device. He was at this point noted to have marked leukocytosis with circulating blasts: WBC 31,000/*μ*L (8% neutrophils, 7% bands, 10% monocytes, 10% metamyelocytes, 20% myelocytes, 14% promyelocytes, and 10% blasts), Hemoglobin 8.4 g/dL, and Platelets 21,000/*μ*L. A bone marrow biopsy demonstrated 65% myeloblasts by morphology. Both morphology and immunophenotype suggested monoblastic features. The patient did not have a FLT3 mutation and cytogenetics demonstrated deletion 16q12 in nine of fourteen metaphases.

At that point, the patient was referred to our center for management of AML. He arrived in a wheelchair and his left leg was markedly swollen. There was a 3 × 3 cm open wound on the medial aspect of his left knee with mild surrounding erythema and some serosanguineous drainage. He was having chills in clinic and was admitted to the hospital for further management. Laboratory studies on admission were notable for a ferritin of 15,162 ng/mL. His WBC cell count was found to be >100,000/*μ*L (predominantly blasts). His clinical status deteriorated rapidly. Within hours, he developed a new oxygen requirement and confusion. Leukapheresis was performed as a temporizing measure to allow family to arrive from out of state. He tolerated the procedure well, and subsequently counts were controlled with hydrea and he stabilized for several days. An investigational therapy was started as part of a clinical trial, but the patient developed respiratory failure from a bacterial pneumonia and passed away two weeks after his admission.

## 3. Discussion

There has been a long history of reports describing a variety of paraneoplastic phenomena associated with myelodysplastic syndromes, particularly those with autoimmune manifestations [3]. Among the disorders that have been previously described include vasculitic skin rash, pericarditis, pyoderma gangrenosum, glomerulonephritis, relapsing polychondritis, Raynaud's phenomenon, polymyalgia rheumatica, Sjogren's syndrome, Coomb's negative hemolytic anemia, idiopathic thrombocytopenic purpura, and chronic inflammatory demyelinating polyneuropathy (reviewed in [3, 4]). In the three cases outlined above, we describe what we believe is yet another paraneoplastic syndrome associated with MDS/MPN, one that heralds aggressive transformation to AML and a particularly poor prognosis. 

The most striking feature of the three cases described above was the presence of unilateral limb swelling that suggested underlying abscess or infection and necessitated surgical exploration. In each case, however, abscess was not encountered upon surgical exploration of the affected area and wound cultures were negative. Skin biopsies were suggestive of inflammation (urticaria in the case of patient 1 and chronic fibrosing panniculitis in the case of patient 3) without convincing evidence of leukemic infiltration. Panniculitis, which involves chronic inflammation and fibrous thickening of subcutaneous adipose tissue, has previously been described in blastic transformation of myelofibrosis [5] and as a presenting symptom of chronic myelomonocytic leukemia [6]. Notably, both patient 1 and patient 3 described here had acute monocytic leukemia, or AML M5, by FAB classification. Magnetic resonance imaging of the affected limb suggested underlying diffuse soft-tissue edema (patient 1) or pyomyositis (patient 2). There also appeared to be a transient, migratory nature to the phenomenon, as both patient 1 (left leg, then right leg) and patient 2 (right leg, then left elbow) had different limbs affected at different times. 

Given the many autoimmune manifestations of myelodysplastic syndromes, it is natural to speculate as to whether the phenomenon described here is yet another autoimmune manifestation of MDS. The presence of strikingly high ferritin levels in all three cases in the absence of evidence of a hemophagocytic syndrome is consistent with a state of acute inflammation. Furthermore, patient 2 responded to high-dose steroids with resolution of her symptoms, which suggests that immune dysregulation played a role in the pathogenesis of the underlying disorder.

The clinical course of all three patients was aggressive. All three patients were too ill to tolerate intensive treatment for leukemia at the time of their acute presentation. All had been hospitalized as inpatients for over a month while their limb swelling was worked up, and all three underwent surgical exploration of their affected limbs that did not yield drainable abscess fluid or improve their condition. In the meantime, their clinical condition and performance status deteriorated and in the case of patient 1 and patient 3, by the time that their blood counts revealed transformation to acute myeloid leukemia, it was too late to initiate intensive treatment. In select cases, however, early intervention with corticosteroids may result in improvement or resolution of the symptoms and permit intensive therapy for AML to be delivered, as was the case with patient 2. Previously, a variety of autoimmune manifestations of MDS have been shown to respond to immunosuppressive therapy, and hematologic responses to steroids have been reported [7].

We suggest that an inflammatory syndrome characterized by limb swelling (often unilateral), soft tissue edema, an associated inflammatory infiltrate, and other signs of inflammation, including striking hyperferritinemia, may herald aggressive transformation of MDS/MPN to AML. Patients with known MDS/MPN who present with these features may benefit from early bone marrow examination to assess disease status and MDS/MPN or AML should be high on the differential diagnosis for patients with abnormal CBCs who newly present with features of this syndrome. In select patients, early intervention with corticosteroids may result in improvement or resolution of the symptoms and permit intensive therapy for AML to be delivered.
